# Radiologische Assistentenfortbildung während der COVID-19-Pandemie: Konzeption und Evaluation eines dreiphasigen onlinebasierten Lehrkonzeptes

**DOI:** 10.1007/s00117-022-01005-7

**Published:** 2022-05-05

**Authors:** Maximilian Thormann, Teresa Lerach, Sebastian Gottschling, Jazan Omari, Maciej Pech, Alexey Surov

**Affiliations:** 1 Klinik für Radiologie und Nuklearmedizin, Universitätsklinikum Magdeburg, Magdeburg, Deutschland; 2 Klinikum Westbrandenburg, Medizinische Hochschule Brandenburg Theodor Fontane, Brandenburg a. d. Havel, Deutschland; 3 Klinik für Radiologie und Nuklearmedizin , Universitätsklinikum Magdeburg, Leipziger Str. 44, 39120 Magdeburg, Deutschland

**Keywords:** Weiterbildung, Radiologie, Medical education, Radiology

## Abstract

Kontaktbeschränkungen und Abstandsregeln haben die klinische Weiterbildung vor große Herausforderungen gestellt. Innovative und interaktive Konzepte sind notwendig, um die Weiterbildung auch in Zeiten von Corona aufrechtzuerhalten. In der aktuellen Literatur sind Lehrkonzepte für Ärztinnen und Ärzte in der Facharztweiterbildung jedoch unterrepräsentiert. In dieser Arbeit wird ein onlinebasiertes Fortbildungskonzept in der Radiologie vorgestellt, das die klassische Fallvorstellung in ein dreiphasiges, interaktives Lernmodell umgestaltet. Dieses besteht aus einer selbstständigen Fallbearbeitung mit Anknüpfung an bestehendes Wissen, gefolgt von einer Falldiskussion und gezielten Fragen sowie Feedback durch die teilnehmenden Oberärztinnen und Oberärzte. Nach 12 Monaten erfolgte eine fragebogenbasierte Evaluation durch die Weiterbildungsassistentinnen und -assistenten. Hierbei zeigte sich, dass eine deutliche Mehrheit der Teilnehmerinnen und Teilnehmer das Fortbildungskonzept positiv hinsichtlich seines Schweregrades, der Repräsentativität für die klinische Tätigkeit sowie der Relevanz für den Facharzt bewerteten. Ebenso ergaben sich Erkenntnisse hinsichtlich der zukünftigen Gestaltung des Konzepts in Bezug auf Dauer, Modalität der zu besprechenden Bildgebungsverfahren und Häufigkeit. Alle Befragten gaben an, vom Format profitiert zu haben. Onlinebasierte Fortbildungskonzepte können demnach einen relevanten Beitrag zur Weiterbildung von Assistenzärztinnen und -ärzten leisten und stellen eine adäquate Alternative bzw. Erweiterung analoger Fortbildungskonzepte dar.

## Abstract (en)

The coronavirus pandemic has posed major challenges to clinical training. Innovative and interactive concepts are needed to maintain training for residents even in times of contact restrictions and distance rules. Training concepts for residents are underrepresented in the current literature. We present an innovative online-based training concept for radiology residents in our department, based on a tripartite design; independent case preparation where residents refer to existing knowledge is followed by a case discussion and specific questions and feedback from the participating senior physicians. A questionnaire-based evaluation of the training concept was carried out after 12 months. We found that participants rated the training concept positively when it came to difficulty, representativeness of clinical work and relevance for specialist training. The questionnaire responses also provided insights into the desired duration, the selection of imaging modalities, and frequency of the concept in the future. All respondents stated that they had benefited from the format. Online-based concepts can therefore be a relevant contribution to the clinical training of residents and represent an adequate alternative to or extension of analog concepts.

Die COVID-19-Pandemie hat nicht nur die studentische Lehre, sondern auch die Weiterbildung für Ärzte und Ärztinnen in der Facharztausbildung vor Herausforderungen gestellt. Kontaktbeschränkungen, Abstandsregeln und Quarantänemaßnahmen haben strukturierte und regelmäßige Fortbildungsveranstaltungen erschwert [1, 15]. Auch die verfügbaren Impfungen haben angesichts wiederholt steigender Inzidenzwerte und einer zunehmenden Hospitalisierungsrate die Maßnahmen der Kontaktbegrenzung vielerorts nicht aufheben können [19]. Für Ärztinnen und Ärzte in der Weiterbildung ist die strukturierte Aneignung fachlicher Kompetenzen als Vorbereitung für den Facharzt und die weitere klinische Laufbahn jedoch von zentraler Bedeutung [7, 9]. Die klinische Routine allein vermag es nicht, eine strukturierte Ausbildung zu vermitteln [1]. Erschwerend kommt hinzu, dass durch die Absage elektiver Untersuchungen das Patientenaufkommen zum Teil deutlich zurückgegangen ist [1, 15]. Ebenfalls ist eine regelmäßige Erhebung des Wissensstandes der Weiterbildungsassistentinnen und -assistenten essenziell. Eine mögliche Alternative ist die digitale Durchführung abteilungsinterner Fortbildungen [2, 8]. Ziel dieser Arbeit ist es, eine wöchentliche digitale Fortbildung in der Radiologie vorzustellen, die auf einem dreiphasigen Konzept beruht. Auf eine selbstständige aktive Fallvorbereitung mit Anknüpfung an bestehendes Wissen folgt eine interaktive Falldiskussion und gezielte Rückfragen sowie Feedback durch die teilnehmenden Oberärzte. Nach 12 Monaten wurde das Konzept hinsichtlich seiner Effektivität und Akzeptanz unter den Assistenzärztinnen und Assistenzärzten evaluiert.

## Studiendesign und Methoden

### Fortbildungskonzept

Zur Aufrechterhaltung der Weiterbildung der Assistenzärztinnen und -ärzte während der COVID-19-Pandemie erfolgte die Konzipierung einer wöchentlichen onlinebasierten Fortbildungsveranstaltung. Hierbei wurde je ein klinischer Fall aus der Schnittbildgebung ausgewählt, der wiederkehrende Untersuchungen oder wichtige Aspekte wie z. B. der Notfalldiagnostik oder der onkologischen Bildgebung repräsentierte. Die Verantwortung für die Vorbereitung der Fortbildung rotierte unter den für die Schnittbildgebung zuständigen Oberärztinnen und Oberärzten. Die Ärztinnen und Ärzte in Weiterbildung erhielten jeweils 2 h vor Beginn der Veranstaltung einen Link zu den Bildern sowie den klinischen Angaben zum Fall zum selbstständigen aktiven Studium. Während dieser Zeit sollten Notizen zu bildmorphologischen Besonderheiten, ihrer Priorisierung sowie zur klinischen Relevanz gemacht werden. Die Fortbildung wurde online über Zoom abgehalten (Zoom Video Communications, San José, CA, USA). Die Dauer der Fortbildung umfasste mindestens 5 und maximal 10 min.

### Auswahl der Fälle

Die Fälle wurden durch die für die Schnittbildgebung zuständigen Oberärztinnen und Oberärzten im Hinblick auf Kenntnis für die tägliche Diagnostik, das Erkennen bestimmter Krankheitsbilder, die klinische Einschätzung weiterer Abklärungen oder die Notfalldiagnostik ausgewählt. Es wurde auf ein ausgewogenes Verhältnis zwischen den einzelnen fachlichen Kategorien als auch zwischen Organsystem geachtet. Es wurden sowohl Bilder aus der CT- als auch aus der MRT-Bildgebung ausgewählt. Den Oberärztinnen und Oberärzten wurde es freigestellt, die Details der Untersuchungen vorab bekanntzumachen oder die Bilder erst zu Beginn der Fortbildung zu präsentieren.

### Ablauf der Veranstaltung

Die Fortbildung folgte einem dreiphasigen Konzept. Nach erfolgtem Selbststudium wählten sich alle anwesenden Assistenzärztinnen und Assistenzärzte zum wöchentlichen Termin in den bereitgestellten Zoom-Link ein. Eine Weiterbildungsassistentin bzw. ein -assistent wurde vom Oberarzt gebeten, der Gruppe seine Befundung des Falles strukturiert vorzustellen, durch das Teilen seines Bildschirmes bildmorphologische Auffälligkeiten für alle anderen zu präsentieren und zu kommentieren. Die die Veranstaltung durchführenden Oberärzte begleiteten die Befundung des Vortragenden kritisch und stellten fallspezifische Rückfragen an den Vortragenden sowie die anderen Teilnehmerinnen und Teilnehmer. Parallel dazu konnte die Chatfunktion des Zoom-Programmes genutzt werden. Zum Ende der Fallbesprechung gab ein Teilnehmer eine Zusammenfassung der wichtigsten epidemiologischen, klinischen und bildmorphologischen Besonderheiten des Falles, unter Verweis auf das erworbene Wissen.

Exemplarisch wird im Folgenden ein Fall aus der Fortbildung vorgestellt. Es handelte sich um eine 63-jährige Patientin, die pulslos und reanimationspflichtig aufgefunden wurde. Der wegweisende klinische Befund lautete Aortendissektion Stanford B mit Hämatotamponade, Rippenserienfraktur nach Reanimation. Die klinischen Angaben wurden 2 h vor der Veranstaltung an alle Weiterbildungsassistenten mitsamt eines Links zu den Bildern zur selbstständigen Vorbereitung versandt. Zu Beginn der Veranstaltung wurde ein Assistent vom Oberarzt aufgefordert, seinen Befund strukturiert zur präsentieren, unter Wahrung der klinischen Relevanz. Für die Präsentation waren 3 min eingeplant. Hiernach gab der Oberarzt Feedback an den vorstellenden Assistenten, gefolgt von Rückfragen an alle Teilnehmenden (z. B. Ätiologie und Einteilung der Aortendissektion, häufige Nebenbefunde nach Reanimation, CT-morphologische Zeichen einer Herzinsuffizienz etc.). Andere klinisch sekundäre Nebenbefunde wie eine vorliegende Hufeisenniere, Nierenzysten sowie „interstitial lung abnormalities“ (ILA) wurden erwähnt und ein weitergehendes Studium in Eigenregie empfohlen. Im Anschluss gab es Raum, mögliche Fragen im Plenum zu klären. Ein Assistent fasste die wichtigsten Inhalte des Falles in eigenen Worten zusammen. Die Assistenten hatten im Anschluss die Möglichkeit, dem Oberarzt kurze Rückmeldung zum Fall zu geben.

### Evaluation

Das onlinebasierte Fortbildungskonzept wurde im November 2020 eingeführt. Nach 12 Monaten wurden die Assistenzärztinnen und Assistenzärzte aufgefordert, an einer Online-Evaluation des Konzeptes teilzunehmen. Der Link zur Befragung wurde per E‑Mail verschickt, die Beantwortung des Fragebogens erfolgte anonym. Mithilfe einer 5‑stufigen Intervallskala von „stimme voll zu“ bis „stimme überhaupt nicht zu“ wurden folgende Themenschwerpunkte erfasst: Schweregrad der Fortbildung, Repräsentativität für den klinischen Alltag sowie Relevanz für die Facharztweiterbildung. Des Weiteren wurde evaluiert, welcher Zeitumfang, welche Frequenz und welcher Schwerpunkt hinsichtlich der spezifischen Schnittbildgebung (MRT vs. CT) aus Sicht der Weiterbildungsassistentinnen und -assistenten gesetzt werden sollte.

## Ergebnisse

### Stichprobe

Zum Zeitpunkt der Befragung waren in unserer Abteilung 18 Assistenzärztinnen und Assistenzärzte beschäftigt. 16 von 18 Weiterbildungsassistentinnen und -assistenten nahmen an der Befragung teil (88,9 %). Aufgrund der hohen Rücklaufquote gehen wir von einer repräsentativen Stichprobe aus.

**Figure 1:**
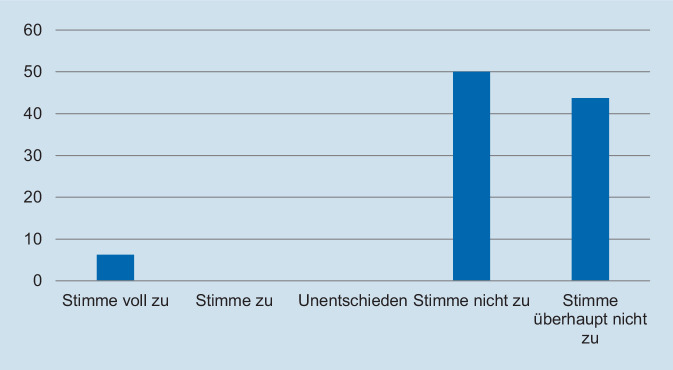

**Figure 2:**
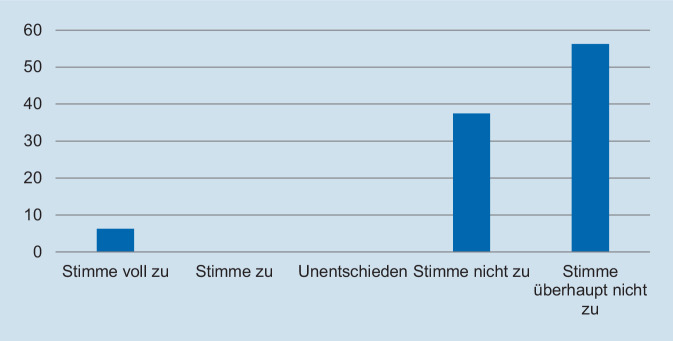

**Figure 3:**
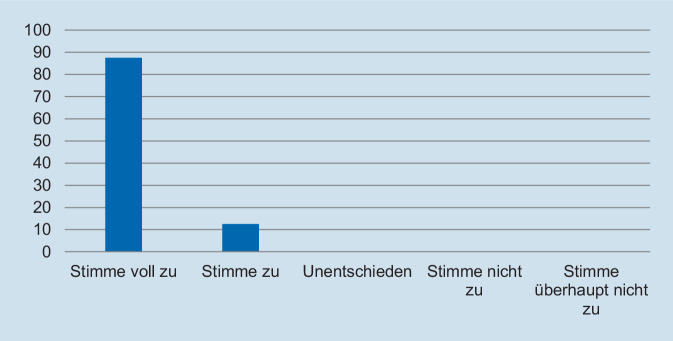

**Figure 4:**
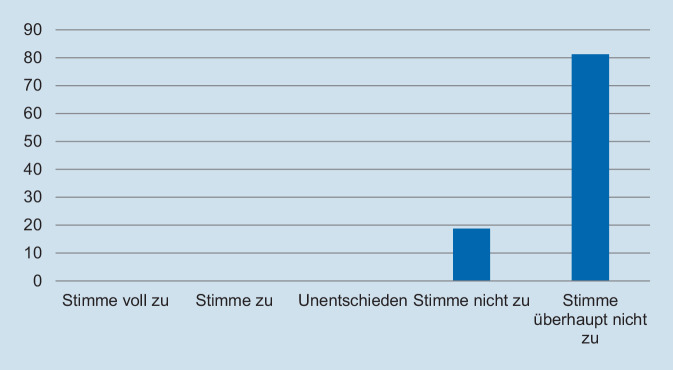

**Figure 5:**
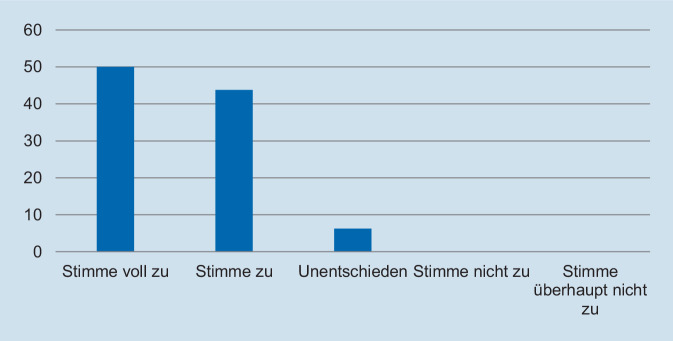

**Figure 6:**
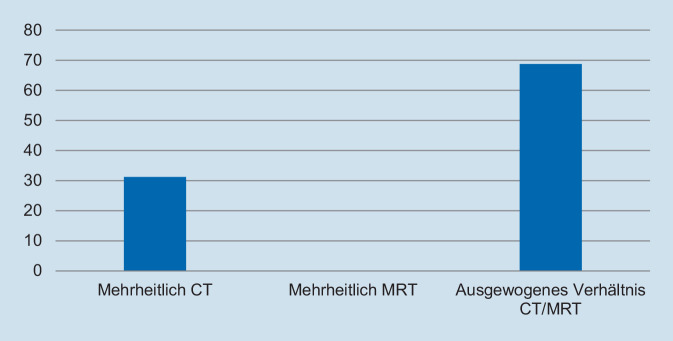

**Figure 7:**
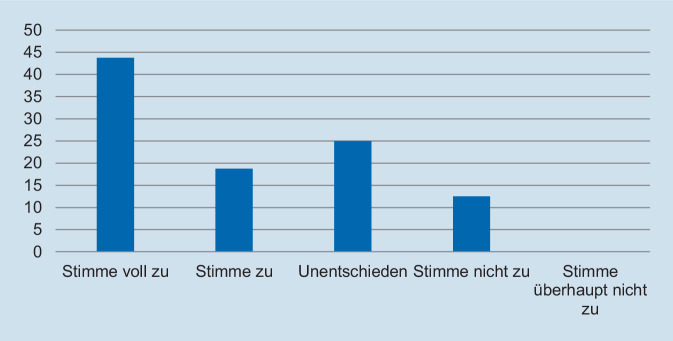

**Figure 8:**
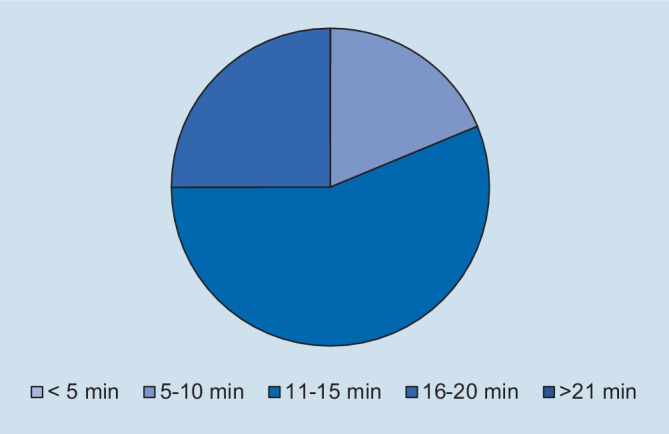

**Figure 9:**
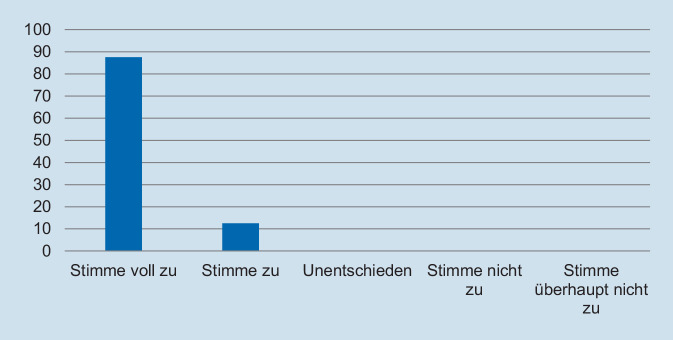

Hinsichtlich des Schweregrades zeigte sich, dass die deutliche Mehrzahl der Assistentinnen und Assistenten die Fallpräsentation als angemessen erachtete. So gaben 93,8 % der Befragten an, dass die Fallpräsentation „nicht“ oder „überhaupt nicht“ zu schwer gewesen sei (Abb. 1). In Bezug auf die Repräsentativität für die klinische Tätigkeit zeigte sich ein ähnliches Bild: Auf die Frage, ob der präsentierte Fall *nicht* repräsentativ für die tägliche Arbeit ist, gaben 93,8 % der Befragten an, dass sie „nicht“ oder „überhaupt nicht“ zustimmten (Abb. 2). Des Weiteren wurde die Relevanz für die Facharztweiterbildung erfragt. Auf die Frage, ob Fallbesprechungen für die Facharztweiterbildung hilfreich sind, gaben alle Befragten an, zuzustimmen oder voll zuzustimmen (Abb. 3). In ähnlicher Weise zeigte sich, dass die Teilnehmerinnen und Teilnehmer von regelmäßigen Fallvorstellungen zu profitieren scheinen. So gaben auf die Frage, ob die sie vom Fall der Woche *nicht* profitierten, alle Befragten an, der Aussage „nicht“ oder „überhaupt nicht“ zuzustimmen (Abb. 4). Der Umfang von Fallpräsentation und -besprechung wurde von 93,8 % als angemessen betrachtet (Abb. 5). Hinsichtlich der behandelten Modalitäten wünschten sich 68,8 % der Teilnehmerinnen und Teilnehmer ein ausgewogenes Verhältnis von CT- und MRT-Bildgebung, während eine überwiegende Behandlung von Fällen aus der CT-Bildgebung von 31,3 % gewünscht wurde (Abb. 6). Eine bevorzugte Besprechung von Fällen aus der MRT wurde nicht präferiert. Zehn Teilnehmerinnen und Teilnehmer (62,5 %) würden sich mehr als einmalige Fallbesprechungen pro Woche wünschen, während nur 2 Befragte (12,5 %) dies verneinten (Abb. 7). Hinsichtlich des zeitlichen Umfanges bevorzugte eine Mehrheit der Teilnehmerinnen und Teilnehmer (56,3 %) eine Dauer von 11–15 min (Abb. 8). Alle an der Umfrage Teilnehmenden wünschten sich eine Fortführung der Lehrveranstaltung (Abb. 9).

## Diskussion

Die COVID-19-Pandemie hat nicht nur viele Abläufe im klinischen Alltag geändert, sondern auch neue Konzepte eingefordert, um die medizinische Lehre für Studenten und Fortbildungsveranstaltungen für Weiterbildungsassistentinnen und -assistenten trotz Distanzregelungen und Quarantänemaßnahmen weiterhin abhalten zu können [14, 17]. In der Radiologie, die auf ein strukturiertes Vorgehen bei der Befundung und auf die Kenntnis möglicher Differenzialdiagnosen angewiesen ist, hat sich dies besonders bemerkbar gemacht [6, 13]. Durch die Absage elektiver Operationen und Untersuchungen hat sich die Breite der durchgeführten Untersuchungen reduziert, um Kapazitäten für Notfallpatienten schaffen zu können [18]. Rotationspläne mussten aufgrund von Vorsichtsmaßnahmen, Infektionen oder Quarantäneregelungen des Personals verschoben werden. Zusätzlich nahm die Dienstbelastung durch erhöhten Krankenstand zu. Junge und fortgeschrittene Weiterbildungsassistentinnen und -assistenten waren in unterschiedlichem Maße betroffen. Insbesondere junge Ärztinnen und Ärzte waren vermehrt auf das Selbststudium angewiesen und befürchteten, unzureichend auf Dienste und Notfallsituationen vorbereitet zu sein [11]. Fortgeschrittene Assistentinnen und Assistenten hatten die Sorge, die geforderte Zahl an Untersuchungen und Interventionen nicht im üblichen Zeitrahmen absolvieren zu können und die Facharztprüfung nicht wie geplant ablegen zu können. Darüber hinaus wurden interne und externe Fortbildungsveranstaltungen, Journal Clubs und Vortragsreihen verschoben oder ganz abgesagt, sodass Kernelemente einer strukturierten Weiterbildung fehlten.

Einige Institutionen sind dazu übergangen, zumindest einen Teil der früheren Präsenzveranstaltungen online abzuhalten. Programme wie Microsoft Teams (Microsoft, Redmont, WA, USA), Cisco Webex (Cisco Systems, San José, CA, USA) oder Zoom sind einige der meistbenutzten Softwarelösungen und ermöglichen es, zumindest einen Teil Lehre in einem interaktiven Rahmen aufrechtzuerhalten [6, 11, 18]. Zusätzliche Funktionen wie Chatfunktion, Handheben und das Erstellen von Live-Umfragen ermöglichen ein akzeptables interaktives Niveau [18]. Meist beinhalten diese Konzepte jedoch lediglich in ein Online-Format umgestaltete passive Präsenzkonzepte, ohne die Möglichkeiten einer aktiven Mitgestaltung zu nutzen.

Die aktive Auseinandersetzung mit dem Lernstoff ist jedoch Kernelement eines nachhaltigen Lernens [10]. In unserer Abteilung erfolgte daher im Sinne eines konstruktivistischen Lernansatzes die Umgestaltung eines ursprünglich auf passiver Wissensvermittlung basierenden Formates – der Fallvorstellung – in eine aktive Wissensvermittlung bzw. Wissenskonstruktion. Durch das dreiphasige Konzept mit selbstständiger Fallvorbereitung mit Rekurrenz auf bestehendes Wissen wurde die aktive Beschäftigung mit dem Lehrinhalt gezielt gefördert. Die Generierung von Wissen wurde durch die anschließende Fallvorstellung und -diskussion gezielt gefördert. Durch Elaboration wurde der Lerninhalt durch die Teilnehmer mit bereits bestehendem Wissen verknüpft und strukturiert [3]. Durch die Betonung des aktiven Aufnehmens des Lerninhaltes, im Gegensatz zum passiven Format der reinen Fallvorstellung, sollten insbesondere Kompetenzen im Bereich des selbstregulierenden Lernens und der Problemlösestrategie erlangt werden. Durch die anschließende Zusammenfassung der wichtigsten Inhalte des Falles im Sinne des „testing effects“ sollten die Kernelemente des erlernten Wissens besser behalten werden [16]. Zudem legen wir einen Schwerpunkt auf das Lernen in der Peergroup. Nicht nur können einzelne Schritte bei der Befundung im offenen Plenum besprochen werden. Die Assistentinnen und Assistenten haben auch die Möglichkeit, am Modell der Fallvorstellung eigenes Wissen zu generieren. Durch alltagsnahe und relevante klinische Fälle ergab sich zudem ein besonderer motivationaler Anreiz.

Die Rückmeldung der Assistenzärztinnen und Assistenzärzte auf das eingeführte Format war dabei mehrheitlich positiv. Sowohl der Schwierigkeitsgrad als auch die Repräsentanz der ausgewählten Fälle wurde für gut befunden. Eine Mehrheit der Teilnehmerinnen und Teilnehmer wünschte sich sogar, dass die Fallbesprechungen mehrfach pro Woche stattfinden, was die Notwendigkeit strukturierter und begleiteter Weiterbildung reflektiert. Stimmen, diese Form der Weiterbildung nicht fortzuführen, gab es nicht. Während sich 5 Teilnehmerinnen und Teilnehmer mehrheitlich Fälle aus der CT-Bildgebung wünschten und 11/16 ein ausgewogenes Verhältnis von CT und MRT bevorzugten, wurde ein Überwiegen von Fällen aus der MRT-Bildgebung von keinem der Befragten präferiert. Dies reflektiert die Bedeutung der CT für die Notfalldiagnostik und ihr Volumen an der täglichen Schnittbildgebung. Die aktuelle Länge der Fallbesprechung wurde dabei von der Mehrheit der Befragten als zu kurz empfunden und eine Verlängerung auf 11–15 min bevorzugt.

Unsere Evaluation zeigt, dass das Konzept von den Weiterbildungsassistentinnen und -assistenten gut angenommen und als nützlich für die eigene Weiterbildung und den eigenen Wissensstand erachtet wird. Insbesondere die Gestaltung der Online-Fortbildung in Form eines virtuellen Raumes ermöglicht hierbei eine kritische Betrachtung der präsentierten Fälle: Durch den aktiven Austausch zwischen Oberärztinnen und Oberärzten und Assistenzärztinnen und -ärzten können etwa Hinweise auf leicht zu übersehende Befunde gegeben werden. Darüber hinaus profitieren die Teilnehmerinnen und Teilnehmer durch den Austausch untereinander im Sinne des sog. „peer-to-peer learning“ [11]. Im Gegensatz zum passiven Aufnehmen von Wissen können beim aktiven generierenden Lernen ggf. gehegte Misskonzepte und häufige Fehler selbstständig identifiziert und verbessert werden. Infolge der aktiven Auseinandersetzung der Weiterbildungsassistentinnen und -assistenten mit den zu befundenden Fällen können darüber hinaus – ganz im Gegensatz zu klassischen Fallvorstellungen – häufige kognitive Verzerrungen wie „the curse of knowledge“ und der „hindsight bias“ (Rückschaufehler) umgangen werden. Einem erfahrenen Dozenten ist es demnach aufgrund seines Wissensvorsprungs schwierig bis unmöglich, die Herausforderungen und Fehlannahmen eines noch unerfahrenen Weiterbildungsassistenten realistisch einzuschätzen [4].

Eine besondere Schwierigkeit von Fortbildungen besteht darin, sowohl für junge als auch für fortgeschrittene Assistenzärztinnen und -ärzte relevante Lehrinhalte zu vermitteln und für beide Gruppen einen ausreichenden Schwierigkeitsgrad zu finden. Dies scheint in unserem Format gelungen. Während der Schwierigkeitsgrad angesichts der überwiegend positiven Antworten sogar noch etwas angehoben werden könnte, gab es keine Stimme, die der Ansicht war, von der Fallpräsentation nicht zu profitieren. Somit ist davon auszugehen, dass sich auch Assistenzärztinnen und -ärzte aus dem vierten und fünften Jahr vom Format angesprochen fühlen. Dies spiegelt sich zudem in der hohen Teilnehmerquote an der Veranstaltung wider.

Ein virtuelles Fortbildungskonzept kann das tägliche klinische Training an der radiologischen Befundungsstation nicht ersetzen, aber sinnvoll ergänzen. Via Screensharing ist es dabei möglich, die Befundung an der PACS-Station zu spiegeln und nicht auf externe DICOM-Viewer-Software zurückgreifen zu müssen. Durch die eigene Vorbereitung der Fälle werden zudem das Selbststudium und die aktive Teilnahme an der Veranstaltung ermutigt. Im Nachgang können die Fälle selbst nachgearbeitet und entsprechende Literatur gelesen werden.

Ergänzt wird die Sitzung in individuellen Fällen durch Live-Umfragen, um den Wissensstand der Teilnehmerinnen und Teilnehmer erheben zu können. Ferner ließe sich unsere Veranstaltung durch Verwendung von Online-Dokumenten wie Google-Docs zur Erfassung und Repetition relevanter Befundungsparameter erweitern. Darauf haben wir zur Simplifizierung des Formats im Rahmen der klinischen Routine und angesichts der Kürze der Veranstaltung bislang verzichtet.

Auch nach der Pandemie werden onlinebasierte Fort- und Weiterbildungsmodelle eine größere Rolle spielen. Sie werden bislang in Präsenz abgehaltene Veranstaltungen entweder ergänzen oder weiterhin ersetzen, sowohl in der studentischen Lehre als auch im Rahmen der Facharztweiterbildung [12]. Hierbei werden sich bestimmte Formate besser zur Digitalisierung eignen als andere. Die Virtualisierung muss dabei nicht zwingend innovativ sein, solange sie den inhaltlichen Bedürfnissen der Assistenzärzte gerecht wird. Es erscheint wichtig, insbesondere die interaktiven Aspekte als auch die Rückkopplung mit den Oberärzten im digitalen Format mit abzubilden [5]. Unsere Evaluation zeigt dabei, wie digitale Lehrformate eingesetzt werden können, um unter erschwerten Bedingungen einen Mehrwert für die Assistenzärztinnen und -ärzte zu schaffen.

Virtuelle Lehrkonzepte sind aus der studentischen Lehre bereits bekannt und haben ihre Vorteile erwiesen. Molwitz et al. geben hier eine Übersicht [12]. Für die Weiterbildung von Assistentinnen und Assistenten ist uns kein ähnliches Konzept mit einer vergleichbaren Evaluation vorliegend. Lehrformen in der Facharztweiterbildung scheinen in der aktuellen Literatur unterrepräsentiert. Auch wenn es sich bei unserer Arbeit nur um eine Stichprobe in einer Abteilung mit einer geringen Zahl an Weiterbildungsassistentinnen und -assistenten handelte, können wir zeigen, dass ein virtuelles strukturiertes Fortbildungskonzept in der Radiologie ohne Weiteres möglich ist und von den Assistenzärztinnen und -ärzten akzeptiert wird. Abteilungen sollten sich bemühen, den Impetus der Pandemie zu nutzen, um onlinebasierte Lehrformate weiter auszubauen und ortsunabhängige Weiterbildung anbieten zu können. Eine Limitation unserer Arbeit ist das Fehlen einer Vergleichsgruppe, die aufgrund der kleinen Kohorte nicht möglich war. Zudem ist ein Vergleich mit anderen Lehrformen in der fachärztlichen Weiterbildung mangels veröffentlichter Studien nicht durchführbar.

In Zukunft soll das Konzept in unserer Klinik auf weitere Bereiche wie die Neuroradiologie und die interventionelle Radiologie ausgedehnt werden. Die Möglichkeiten der digitalen Präsentation von Fällen – auch Live-Streaming ist dabei möglich – sind vielfältig. Auch die Einbindung anderer Fächer, z. B. der Chirurgie, zur umfangreichen Besprechung eines Falles mit seinen klinischen und radiologischen Aspekten ist im digitalen Format leicht durchzuführen. Regelmäßige digitale Wissenskontrollen können den Kompetenzfortschritt der Ärztinnen und Ärzte dabei dokumentieren. Praktische Fertigkeiten wie z. B. die Sonographie sollten dabei jedoch nicht außer Acht gelassen werden.

## References

[1] Alvin MD, George E, Deng F (2020). The impact of COVID-19 on radiology trainees. Radiology.

[2] Chong A, Kagetsu NJ, Yen A, Cooke EA (2020). Radiology residency preparedness and response to the COVID-19 pandemic. Acad Radiol.

[3] Craik FI, Tulving E (1975). Depth of processing and the retention of words in episodic memory. J Exp Psychol Gen.

[4] Glaser C (2019). Fluch des Wissens. Risiko im Manag.

[5] Hempel G, Weissenbacher A, Stehr SN (2021). COVID-19: eine Chance zur Digitalisierung der Lehre?. Anaesthesist.

[6] Klontzas ME, O’Malley E, Afat S (2021). Impact of COVID-19 on radiology education in Europe: a survey by the ESR Radiology Trainees Forum (RTF). Insights Imaging.

[7] Kolokythas O, Patzwahl R, Straka M, Binkert C (2016). Evaluation in der Assistenzarztausbildung. Radiologe.

[8] Marcoux JT (2020). Training residents during the Covid-19 pandemic. J Foot Ankle Surg.

[9] Martin A, Lang E, Ramsauer B (2020). Kontinuierliche medizinische Fortbildung in der Dermatologie für Ärzte und Studierende während der Coronavirus-Pandemie – eine große Herausforderung. J Dtsch Dermatol Ges.

[10] McDaniel MA, Howard DC, Einstein GO (2009). The read-recite-review study strategy: effective and portable: research article. Psychol Sci.

[11] McRoy C, Patel L, Gaddam DS (2020). Radiology education in the time of COVID-19: a novel distance learning workstation experience for residents. Acad Radiol.

[12] Molwitz I, Othman A, Brendlin A (2021). Digital teaching with, during and after COVID-19. Radiologe.

[13] Nadgir R (2020). Teaching remotely: educating radiology trainees at the workstation in the COVID-19 era. Acad Radiol.

[14] Offergeld C, Ketterer M, Neudert M (2021). „Online from tomorrow on please“: comparison of digital framework conditions of curricular teaching at national university ENT clinics in times of COVID-19: Digital teaching at national university ENT clinics. HNO.

[15] Rana T, Hackett C, Quezada T (2020). Medicine and surgery residents’ perspectives on the impact of COVID-19 on graduate medical education. Med Educ Online.

[16] Roediger HL, Karpicke JD (2006). Test-enhanced learning: taking memory tests improves long-term retention. Psychol Sci.

[17] Rose S (2020). Medical student education in the time of COVID-19. J Am Med Assoc.

[18] Virarkar M, Jensen C, Javadi S (2020). Radiology education Amid COVID-19 pandemic and possible solutions. J Comput Assist Tomogr.

[19] Coronavirus COVID-19 global cases by Johns Hopkins CSSE. https://www.arcgis.com/apps/dashboards/bda7594740fd40299423467b48e9ecf6. Zugegriffen: 28. Nov. 2021

